# Heterostructured g-CN/TiO_2_ Photocatalysts Prepared by Thermolysis of g-CN/MIL-125(Ti) Composites for Efficient Pollutant Degradation and Hydrogen Production

**DOI:** 10.3390/nano10071387

**Published:** 2020-07-16

**Authors:** Batukhan Tatykayev, Bilel Chouchene, Lavinia Balan, Thomas Gries, Ghouti Medjahdi, Emilien Girot, Bolat Uralbekov, Raphaël Schneider

**Affiliations:** 1 CNRS, LRGP, Université de Lorraine, F-54000 Nancy, France; b.tatykayev@gmail.com (B.T.); bilel.chouchene@univ-lorraine.fr (B.C.); emilien.girot@univ-lorraine.fr (E.G.); 2 Department of General and Inorganic Chemistry, Al-Farabi Kazakh National University, Al-Farabi Av., 71, Almaty 050040, Kazakhstan; bulat.ural@gmail.com; 3 Conditions Extrêmes et Matériaux: Haute Température et Irradiation, UPR 3079 CNRS, Site Haute Température, 1D avenue de la Recherche Scientifique, 45071 Orléans, France; lavinia.balan@cnrs-orleans.fr; 4 CNRS, IJL, Université de Lorraine, F-54000 Nancy, France; thomas.gries@univ-lorraine.fr (T.G.); ghouti.medjahdi@univ-lorraine.fr (G.M.)

**Keywords:** graphitic carbon nitride, MIL-125(Ti), titanium dioxide, heterostructured photocatalysts

## Abstract

Photocatalysts composed of graphitic carbon nitride (g-CN) and TiO_2_ were efficiently prepared by thermolysis of the MIL-125(Ti) metal organic framework deposited on g-CN. The heterojunction between the 12 nm-sized TiO_2_ nanoparticles and g-CN was well established and the highest photocatalytic activity was observed for the g-CN/TiO_2_ (3:1) material. The g-CN/TiO_2_ (3:1) composite exhibits high visible light performances both for the degradation of pollutants like the Orange II dye or tetracycline but also for the production of hydrogen (hydrogen evolution rate (HER) up to 1330 μmolh^−1^g^−1^ and apparent quantum yield of 0.22% using NiS as a cocatalyst). The improved visible light performances originate from the high specific surface area of the photocatalyst (86 m^2^g^−1^) and from the efficient charge carriers separation as demonstrated by photoluminescence, photocurrent measurements, and electrochemical impedance spectroscopy. The synthetic process developed in this work is based on the thermal decomposition of metal organic framework deposited on a graphitic material and holds huge promise for the preparation of porous heterostructured photocatalysts.

## 1. Introduction

The assembly of semiconductors into heterostructures has recently attracted considerable attention due to the numerous photocatalytic applications of these materials like detoxification of water or solar-to-chemical energy conversion [1,2,3,4,5]. In heterostructured photocatalysts, both the efficient spatial separation of charge carriers and the enhanced light absorption allow to boost the catalytic activity compared to the individual components due to a synergistic effect.

Titanium dioxide TiO_2_ is one of the most important photocatalyst and has been widely used for environmental applications or water splitting [6,7,8,9,10,11]. However, the photocatalytic performances of TiO_2_ are limited by its wide bandgap energy (ca. 3.2 eV) which restricts its photoactivation to UV light and by its modest charge transport efficiency. Graphitic carbon nitride (g-CN) is an organic layered semiconductor composed of carbon and nitrogen with an energy bandgap of ca. 2.7 eV and exhibiting high chemical, thermal and photostability [1,12,13,14]. As TiO_2_, bulk g-CN suffers from several drawbacks including the fast recombination of photogenerated charge carriers and a low specific surface area (less than 10 m^2^g^−1^), which decrease its catalytic performance [1,12,13,14]. However, the conduction band of g-CN is located at ca. −1.3 eV at pH 7 vs. NHE and is more negative than that of TiO_2_ [15,16], which allows photogenerated electrons to be efficiently transferred from the conduction band of g-CN to the conduction band of TiO_2_. This decrease of the photogenerated electron-hole pair’s recombination allows significant improvements in the photocatalytic performance of g-CN/TiO_2_ composites.

Various methods have been developed for the preparation of g-CN/TiO_2_ heterostructured photocatalyts. The most commonly used process is the ultrasonication or the mechanical mixing of pre-formed TiO_2_ and g-CN followed by a heat treatment at temperatures varying from 300 to 500 °C to hybridize their energy levels [17,18,19,20,21]. To improve the interfacial connection between TiO_2_ and g-CN and thus the delocalization of charge carriers, synthetic methods involving the deposition of Ti precursors like Ti(On-Bu)_4_ at the surface of exfoliated g-CN followed a hydro- or a solvothermal reaction and a calcination have been reported [22,23,24,25]. The reverse process including the generation of g-CN by calcination of N-rich precursors (melamine and/or cyanuric acid) in the presence of TiO_2_ nanoparticles or titanate nanotubes has also been investigated [26,27,28,29,30,31,32]. Finally, the vapor phase deposition of g-CN at the surface of TiO_2_ [33] or the surface modification of g-CN with chloroacetic acid to increase the association with TiO_2_ have also been reported [34].

In recent years, the preparation of porous metal oxides derived from metal organic frameworks (MOFs) has gained high interest for environmental and energy-related applications. After thermolysis, the textural properties of MOFs are partially transferred to the porous metal oxide exposing a high density of active sites and in which charge carriers easily diffuse to the surface of the photocatalyst [35]. Using the NH_2_-MIL-125(Ti) as starting MOF, this strategy has successfully been used to prepare photocatalysts associating TiO_2_ nanoparticles deposited on carbon or associated to MoS_2_ [36,37,38]. MIL-125(Ti) is a MOF responding to the formula Ti_8_O_8_(OH)_4_[O_2_C-C_6_H_4_-CO_2_]_6_ and exhibiting a well-defined porosity and high specific surface area (ca. 1700 m^2^g^−1^) [39]. MIL-125(Ti) is a good precursor of TiO_2_ nanocrystals [40,41] that demonstrated a good photocatalytic activity for CO_2_ reduction, As(III) oxidation or pollutant degradation [42,43,44,45,46,47,48].

Herein, a novel synthetic process using in the key step the thermolysis of g-CN/MIL-125(Ti) assemblies was developed to prepare highly active g-CN/TiO_2_ photocatalysts. The mass ratio of g-CN to TiO_2_ was varied and the highest photocatalytic activity was obtained for the g-CN/TiO_2_ (3:1) material. The g-CN/TiO_2_ photocatalyst was not only demonstrated to be highly efficient for the degradation of pollutants in aqueous phase but also for H_2_ photoproduction using NiS as co-catalyst.

## 2. Materials and Methods

### 2.1. Chemicals

Terephthalic acid (98%, Sigma), titanium (IV) propoxide (98%, Sigma), melamine (99%, Sigma), dimethylformamide (DMF) (99.9%, Sigma), Orange II sodium salt (microscopy grade, Sigma), tetracycline hydrochloride (Bioreagent, Sigma), glycerol (>99.5%, Sigma), methanol (99.9%, Sigma) and ethanol (anhydrous, Sigma) were used as received.

### 2.2. Synthesis of MIL-125(Ti)

Terephthalic acid (3 g, 18.28 mmol) was dissolved in 54 mL of anhydrous DMF and 6 mL MeOH by magnetic stirring for 15 min. To the obtained solution was added titanium (IV) propoxide (3.6 mL, 13.08 mmol) and the mixture was stirred for 30 min under argon. Then, the solution was transferred into a 100 mL Teflon-lined stainless autoclave and heated in an oven at 150 °C for 48 h. After cooling to room temperature, MIL-125(Ti) particles were collected by centrifugation (4000 rpm for 15 min), washed with DMF (3 × 30 mL), with MeOH (3 × 30 mL) and finally dried at 70 °C overnight. A typical synthesis affords 1.86 g of a white powder.

### 2.3. Synthesis of Bulk g-CN

Melamine (5 g) was placed into a covered ceramic crucible and heated under air at 500 °C at a rate of 10 °C min^−1^ and then maintained at 500 °C for 2 h. The yellow powder obtained (1.75 g) was ground in an agate mortar. 

### 2.4. Exfoliation of g-CN

Bulk g-CN (0.5 g) was dispersed in 80 mL water and ultrasonicated for 60 min using a Sonic Dismembrator 550 (Fisher Scientific, Illkirch, France). Next, the obtained white-yellow dispersion was centrifuged for 30 min at 5000 rpm, the precipitate collected, washed with EtOH (30 mL) and dried at 70 °C overnight.

### 2.5. Preparation of g-CN/TiO_2_ Photocatalysts

A representative synthesis of the g-CN/TiO_2_ (3:1) photocatalyst is described. Exfoliated g-CN (180 mg) and MIL-125(Ti) (120 mg) were dispersed in 5 mL water and the mixture sonicated for 30 min. After centrifugation (4000 rpm for 15 min), the powder was washed with EtOH (30 mL) and dried at 70 °C for 3 h. Then, the powder was placed into a covered ceramic crucible, heated under air at 450 °C at a rate of 10 °C min^−1^ and maintained at 450 °C for 2 h before cooling. The g-CN/TiO_2_ composites were used without further treatment for photocatalytic experiments.

### 2.6. Photocatalytic Degradations

A 300 W Osram lamp coupled with UV cut-off filter (λ > 420 nm) was used as light source for photocatalytic degradation experiments. In a typical photocatalytic experiment, 20 mg of photocatalyst was dispersed in 40 mL of the pollutant aqueous solution (Orange II or tetracycline hydrochloride, 10 mg/L) and the mixture was stirred in the dark for 60 min to reach the adsorption-desorption equilibrium. Next, visible light was turned on and at regular irradiation time intervals, 2 mL of the dispersion were withdrawn, centrifuged for 5 min at 15,000 rpm and analyzed by UV-visible absorption to monitor the degradation of the pollutant (485 nm for Orange II and 357 nm for tetracycline).

Scavenging experiments were conducted using the same synthetic protocol except that *tert*-butanol (*t*-BuOH), *p*-benzoquinone (*p*-BQ), ammonium oxalate (AO), sodium azide (NaN_3_) and dimethylsulfoxide (DMSO) were added to the aqueous dispersions before turning on light.

### 2.7. Photocatalytic H_2_ Production

For photocatalytic H_2_ production, the surface of nanocomposites was decorated with NiS nanoparticles using Ni(NO_3_)_2_ and Na_2_S as the precursors for NiS. In a typical experiment, 400 mg of the g-CN/TiO_2_ (3:1) catalyst was dispersed in 45 mL of deionized water and 1.3 mL of a 0.05 M Ni(NO_3_)_2_ was added. The mixture was stirred for 20 min to absorb Ni^2+^ ions at the surface of the g-CN/TiO_2_ (3:1) composite. Next, 1.5 mL of a 0.05 M Na_2_S solution was added to the solution to form NiS. The obtained g-CN/TiO_2_ (3:1)/NiS composite contains ca. 1.5 wt.% NiS and is noted g-CN/TiO_2_ (3:1)/1.5%NiS. The association of NiS with the other g-CN/TiO_2_ photocatalysts was conducted using the same synthetic protocol. 

The photocatalytic hydrogen production was conducted in an outer irradiation type quartz reactor using a 300 W Xe lamp as the light source. A circulation of water with an external cooling coil was used to maintain the temperature of the dispersion at 25 °C. In order to remove dissolved oxygen, the dispersion was first bubbled with nitrogen for 60 min before light irradiation. The reactor was connected to a pure nitrogen gas flow (100 mL min^−1^) used to displace hydrogen from the photoreactor toward the micro gas chromatograph (Varian 490-GC). In a typical experiment, 25 mg of photocatalyst was dispersed in 50 mL aqueous solution containing 10 vol.% of glycerol used as sacrificial substrate and the mixture was stirred to ensure a uniform irradiation of the catalyst dispersion.

### 2.8. Apparent Quantum Efficiency (AQE)

The apparent quantum efficiency (AQE) was determined using the previously described experimental setup and with the Equation (1):

(1) $$AQE=\frac{n\times \Delta G}{I}\times 100\%=\frac{n\times \Delta G}{W\times S\times t}\times 100\%$$

where *n* is the number of H_2_ molecules evolved, ΔG = 237 kJmol^−1^ is the energy needed for one water molecule to entirely split into H_2_ and O_2_, *W* is the power of the lamp, *S* is the irradiated area, and *t* is the reaction time [33]. 

### 2.9. Photocatalyst Characterization

The morphology and the microstructure were investigated by scanning electron microscopy (SEM, Scanning Electron Microscope JSM-6490 LV, JEOL, Croissy, France) and transmission electron microscopy (TEM, Philips CM200 instrument operating at 200 kV, Philips, Suresnes, France). TEM images were acquired by placing a drop of the particles in water onto a gold grid.

X-ray powder diffraction (XRD) patterns were recorded using a Panalytical X’Pert Pro MPD diffractometer using Cu Kα radiation (λ = 0.15418 nm).

X-ray photoelectron spectroscopy (XPS) analyses were carried out on a Gammadata Scienta (Uppsala, Sweden) SES 200-2 spectrometer under ultra-high vacuum (P < 10^−9^ mbar).

Thermogravimetric analysis (TGA) was conducted under O_2_ atmosphere using a TGA/DSC1 STAR equipment (Mettler-Toledo). Measurements were conducted from room temperature to 1000 °C at a heating rate of 10 °C min^−1^.

The Brunauer–Emmett–Teller (BET) specific surface areas were measured with a Micromeritics ASAP 2420 instrument at liquid nitrogen temperature. Before analysis, the samples were out-gassed overnight in vacuum at 40 °C followed by 4 h out-gassing on the analysis port. The micropore volume was determined using the Barrett–Joyner–Halenda (BJH) method. 

A Total Organic Carbon analyzer (Shimadzu TOC-VCSH, Marne-la-Vallée, France) was used to evaluate the amount of dissolved organic carbon before and after the photodegradation reactions.

Fourier transform infrared (FT-IR) spectra were recorded using a Brucker ALPHA spectrometer in the frequency range of 4000–450 cm^−1^. UV-visible absorption spectra of liquid samples were measured using a Thermo Scientific Evolution 220 UV-visible spectrophotometer. The UV-visible diffuse reflectance spectra (DRS) were recorded on a Shimadzu 2600-2700 UV-visible spectrophotometer. Photoluminescence (PL) emission spectra were recorded at room temperature on a Horiba Fluoromax-4 Jobin Yvon spectrofluorimeter. Raman spectra were measured on a Horiba Scientific Xplora spectrometer using 532 nm wavelength incident YAG laser light.

The photoelectrochemical performance of the catalysts was evaluated in a three electrode cell using FTO glass coated with g-CN, TiO_2_ or g-CN/TiO_2_ composites with a surface area of 1 cm^2^ as the working electrode, a Pt wire as the counter electrode and a saturated Ag/AgCl electrode as reference electrode. The electrolyte used was a 0.1 M Na_2_SO_4_ aqueous solution. The photocurrent densities were determined by measuring the current under 100 mW cm^−2^ light illumination provided by a 300 W Xe arc lamp equipped with an AM 1.5G filter. A SP150 BioLogic potentiostat was used to measure the photocurrent response at a constant potential of +0.30 V vs. the reference electrode.

## 3. Results

### 3.1. Photocatalysts Synthesis and Characterization

**Figure 1:**
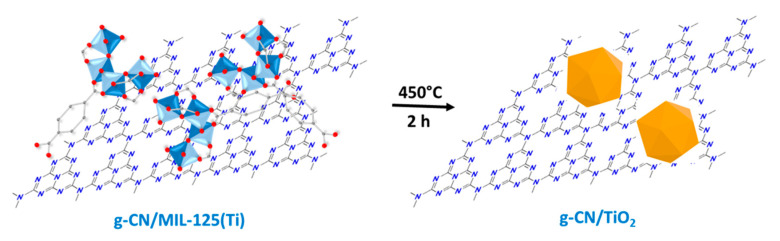
Schematic illustration of the preparation of the graphitic carbon nitride (g-CN)/TiO_2_ photocatalysts from g-CN/MIL-125(Ti) composites.

MIL-125(Ti) particles were produced from Ti(On-Pr)_4_ and terephthalic acid in a DMF/MeOH mixture [39] and associated to exfoliated g-CN under sonication. Next, the g-CN/MIL-125(Ti) composites were calcined at 450 °C for 2 h to decompose MIL-125(Ti) into TiO_2_ (Figure 1). A weight loss of ca. 50% was observed during the thermolysis of pure MIL-125(Ti). g-CN/MIL-125(Ti) composites with g-CN:MIL-125(Ti) weight ratios of 1:2, 2:2, 3:2, 4:2, and 8:2 were prepared to engineer g-CN/TiO_2_ photocatalysts with g-CN:TiO_2_ ratios of 1:1, 2:1, 3:1, 4:1, and 8:1, respectively.

**Figure 2:**
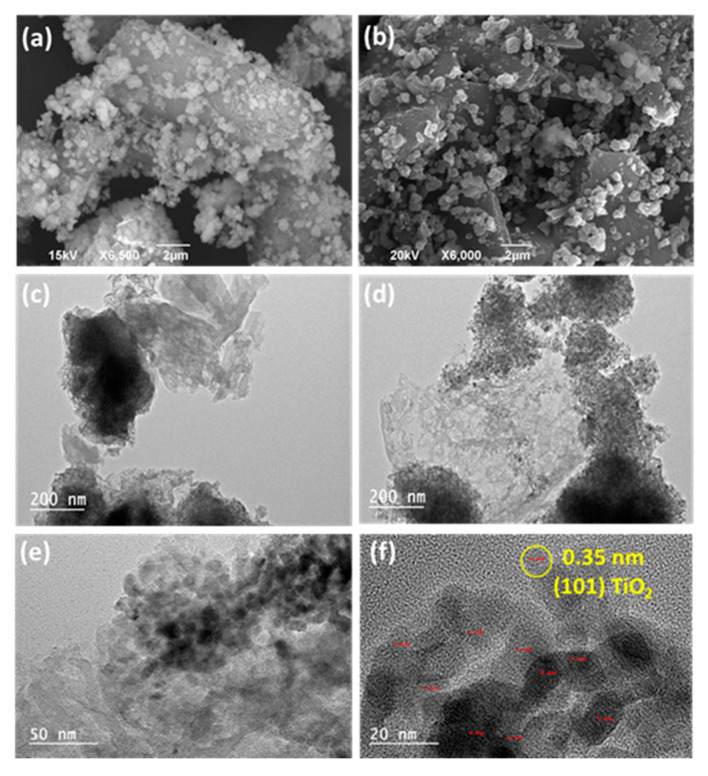
Scanning electron microscopy (SEM) images of (**a**) g-CN/MIL-125 (3:2) and (**b**) g-CN/TiO_2_ (3:1), transmission electron microscopy (TEM) images of (**c**) g-CN/MIL-125 (3:1), and (**d**–**f**) g-CN/TiO_2_ (3:1) composites.

The morphology, the structure and the composition of g-CN/MIL-125(Ti) and g-CN/TiO_2_ composites were first investigated by SEM and TEM (Figure 2). After exfoliation by sonication, g-CN sheets have sizes varying from a few hundred of nanometers up to several micrometers and exhibit a crumpled layered morphology with a relatively smooth surface (Figure 2a). Using g-CN/MIL-125 (3:2) and g-CN/TiO_2_ (3:1) samples as representatives, MIL-125(Ti) and TiO_2_ particles are bound to g-CN sheets and uniformly distributed at their surface as indicated by SEM, TEM, TEM-associated EDS analyses and by the corresponding elemental mappings (Figure 2a–d, Figures S1 and S2). A marked exfoliation of g-CN can also be observed by SEM during the thermolysis of MIL-125(Ti) particles, which will be confirmed by BET specific surface measurements (*vide infra*). The SEM images show that MIL-125(Ti) particles decompose into TiO_2_ particles with an average diameter of ca. 400 nm (Figure 2a,b) but TEM analyses demonstrate that TiO_2_ is actually made up of clusters composed of TiO_2_ nanoparticles with an average size of 12 ± 6 nm (Figure 2e). The HR-TEM image of these TiO_2_ nanocrystals shows a lattice spacing of 0.35 nm, which corresponds to the (101) plane of anatase TiO_2_ (Figure 2f).

**Figure 3:**
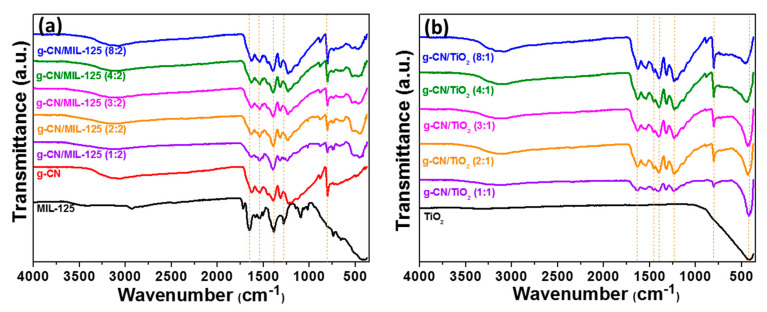
Fourier transform infrared (FT-IR) spectra of (**a**) MIL-125(Ti), g-CN, and of g-CN/MIL-125(Ti) composites and (**b**) TiO_2_ and g-CN/TiO_2_ composites.

The FT-IR spectrum of MIL-125(Ti) exhibits the typical vibrational bands between 1715 and 1300 cm^−1^ corresponding to the C=C and C-H bonds of the aromatic ring and to the asymmetric (1648 cm^−1^) and symmetric (1386 cm^−1^) stretching vibrations of the carboxylate functions (Figure 3a). The signals observed at 807, 739, and 657 cm^−1^ can be assigned to Ti-O-Ti-O vibrations [49]. For pure g-CN, the peaks located from 1658 to 1236 cm^−1^ correspond to the stretching modes of the carbon-nitrogen (C=N and C-N) bonds, while the sharp peak located at 806 cm^−1^ can be attributed to the out-of-plane breathing vibration of the s-triazine units [15,16,50]. The broad absorption bands at 3235 and 3154 cm^−1^ correspond to the stretching modes of NH_2_ and NH functions. All these signals can be observed in the FT-IR spectra of g-CN/MIL-125(Ti) composites, indicating the successful deposition of MIL-125(Ti) at the surface of g-CN. Pure TiO_2_ generated by the thermal decomposition of MIL-125 only exhibits a strong signal at 410 cm^−1^ corresponding to the vibration mode of Ti-O-Ti bonds in TiO_2_ (Figure 3b) [51]. No signals of organics could be observed indicating that MIL-125 was efficiently decomposed into TiO_2_ by heating at 450 °C for 2 h. The shift observed for the Ti-O bond from 412 to 460 cm^−1^ when increasing the g-CN:TiO_2_ ratio from 1:1 to 8:1 suggests a strong interaction between TiO_2_ and g-CN, which should favor the photocatalytic activity of these materials.

**Figure 4:**
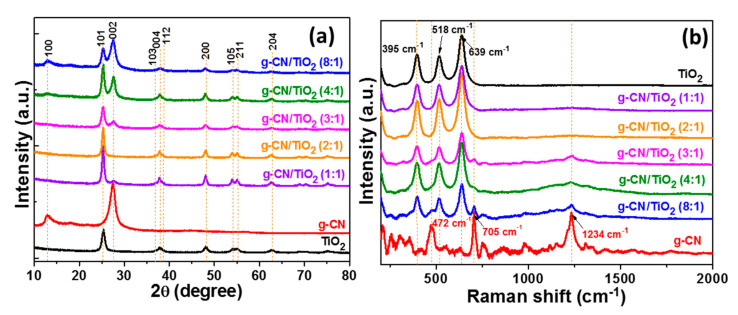
(**a**) X-ray powder diffraction (XRD) patterns and (**b**) Raman spectra of TiO_2_, g-CN, and g-CN/TiO_2_ composites.

XRD and Raman spectroscopy were used to analyse the structure of g-CN/TiO_2_ composites. As previously observed after the pyrolysis of MIL-125(Ti) at a relatively mild temperature, the pure anatase phase of TiO_2_ is formed (Figure 4a) [42,44]. The peaks at 2θ values of 25.35, 36.83, 37.93, 38.72, 48.16, 54.19, 55.16, and 62.75° belong to the (101), (103), (004), (112), (200), (105), (211), and (204) lattice planes of anatase (JCPDS No 21-1272). For g-CN, the diffraction peaks located at 13.03° and 27.51° are related to the (100) interplanar structural packing (repeated s-triazine units) and to the (002) interlayer stacking structure [15,16]. Compared to bulk g-CN, a slight decrease of the cristallite size from 7.4 to 6.9 nm (Figure S3) and a slight shift to higher angles of the (002) peak (Figure S4) were observed for exfoliated g-CN. The intensity of the g-CN related peaks increases with the g-CN content of the nanocomposites. Moreover, a shift of the (002) reflection from 27.51° to 27.66° is observed after the thermolysis of MIL-125(Ti), which confirms the strong interaction of TiO_2_ nanoparticles with g-CN sheets. The Raman spectrum of pure TiO_2_ shows peaks at 395, 518, and 639 cm^−1^ corresponding to the *B1g*, *A1g* + *B1g* and *Eg* modes of anatase TiO_2_, respectively (Figure 4b) [52]. Despite the modest resolution of the g-CN Raman spectrum using the 532 nm laser, three strong peaks corresponding to the vibration of s-triazine units can be observed at 472, 705, and 1234 cm^−1^ [53]. The Raman signals of TiO_2_ and g-CN can all be observed in g-CN/TiO_2_ composites, further confirming the structure of these materials.

**Figure 5:**
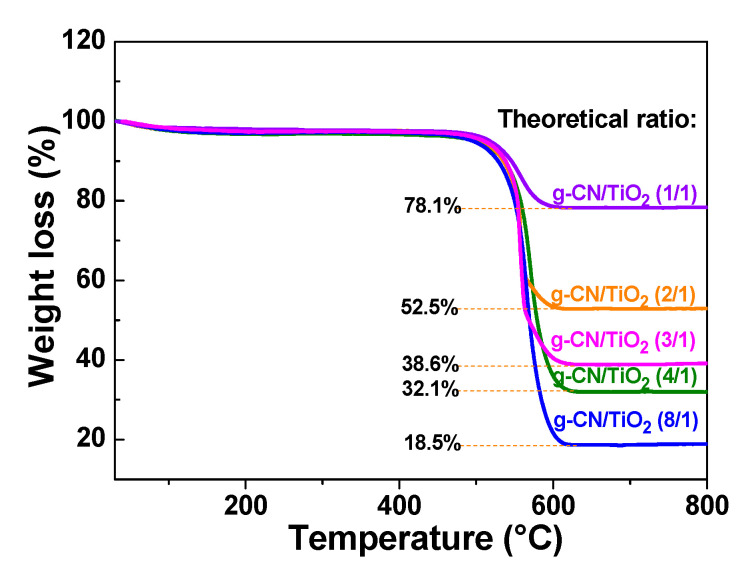
Thermogravimetric curves of g-CN/TiO_2_ composites.

The actual weight ratio of TiO_2_ in g-CN/TiO_2_ composites and the thermal stability of these photocatalysts were determined by TGA. The TGA profiles of the g-CN/TiO_2_ photocatalysts recorded in air atmosphere with a heating rate of 10 °C min^−1^ are given in Figure 5. The weak weight loss (ca. 2%) observed below 100 °C originates from the removal of water molecules adsorbed at the surface of the catalysts. As can be seen from Figure 5, g-CN starts to decompose at ca. 550 °C into CO_2_ and NO_2_. A sharp weight loss is observed at ca. 580 °C for all composites. The content in g-CN was determined from the remaining TiO_2_ weight and was found to be significantly lower than the theoretical ratio (Table 1). The relative content in g-CN is the lowest for composites with a high loading in TiO_2_ (g-CN/TiO_2_ ratios of 1:1 and 2:1), suggesting that TiO_2_ promotes the thermal decomposition of g-CN.

X-ray photoelectron spectroscopy (XPS) was used to investigate the composition and the surface chemical states of C, N, Ti and O elements present in the g-CN/TiO_2_ (3:1) composite (Figure S5). The major signal in the XPS high resolution C 1s spectrum is located at 288.08 eV and can be assigned to sp^2^-hybridized C atoms in nitrogen heterocycles (Figure S6a). The weaker signal observed at 284.93 eV originates from sp^2^-hybridized C-C bonds [54,55]. The N 1s spectrum can be deconvoluted into three peaks at 401.15 eV (N-N bonding structure), 400.09 eV (tertiary nitrogen atoms, N-(C)_3_) and 398.54 eV (sp^2^-hybridized N in triazine rings, C-N=C) (Figure S6b) [55]. The weak signal observed at 404.51 eV can be attributed to charging effects. For Ti 2p, two signals located at 458.87 eV and 464.62 eV corresponding to Ti 2p_3/2_ and Ti 2p_1/2_, respectively, can be observed (Figure S6c). The doublet splitting energy of Ti 2p is of 5.75 eV, which confirms that Ti is in the +4 oxidation state [56]. Finally, the O 1s spectrum can be deconvoluted into two peaks at 530.11 and 532.30 eV corresponding to lattice oxygen atoms (Ti-O bonds) and to carbonate species adsorbed at the surface of the photocatalyst, respectively (Figure S6d).

**Figure 6:**
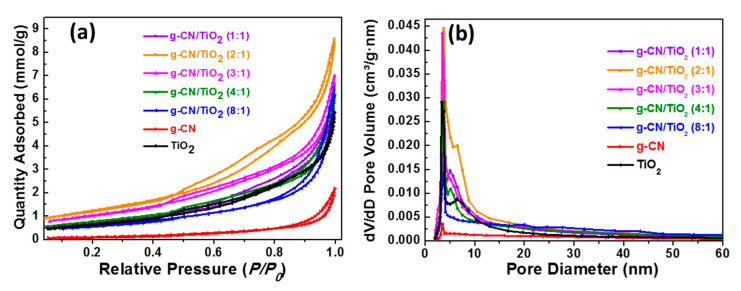
(**a**) Nitrogen adsorption-desorption isotherms of TiO_2_, g-CN, and g-CN/TiO_2_ photocatalysts and (**b**) the corresponding pore-size distributions.

The BET specific surface areas and the porosities of g-CN, TiO_2_, and g-CN/TiO_2_ photocatalysts were investigated by nitrogen adsorption–desorption experiments. Figure 6a shows that all materials exhibit isotherms of type IV according to the Brunauer–Deming–Deming–Teller (BDDT) classification, indicating the presence of mesopores. The hysteresis loops observed at high relative pressure (*P/P*_0_ > 0.8) suggest the presence of slit-like pores, which is consistent with the morphology of the photocatalysts determined by SEM and TEM (Figure 2). Pure g-CN exhibits a low BET specific surface area (9.8 m^2^g^−1^), which is in accordance with previous reports (Table 2) [15,16]. The BET specific surface area of pure TiO_2_ is significantly higher (56.3 m^2^g^−1^), indicating that the textural properties of MIL-125(Ti) were partly transferred to TiO_2_ after the calcination step. The BET specific surface areas of g-CN/TiO_2_ composites increase with the content in g-CN until the g-CN:TiO_2_ ratio of 2:1 and then decrease, which suggests than an optimal amount of MIL-125(Ti) favours the exfoliation of g-CN during the heating at 450 °C and thus the increase of the specific surface area. This may originate from the production of gases like CO_2_ during the thermolysis of MIL-125(Ti) that generate more larger pores that allow to increase the BET specific surface area. The gradual decrease of S_BET_ when increasing the g-CN:TiO_2_ ratio from 3:1 to 8:1 may originate from a pore blocking of TiO_2_ by g-CN. The S_BET_ values measured for g-CN/TiO_2_ composites prepared via annealing of Ti(On-Bu)_4_ in the presence of g-CN or by calcination of melamine in the presence of TiO_2_ crystals typically range from 34 to 77 m^2^g^−1^ [19,22,23,26,28]. The specific surface areas of g-CN/TiO_2_ (2:1 and 3:1) photocatalysts are significantly higher (ca. 102 and 86 m^2^g^−1^) suggesting that during the calcination step, g-CN:MIL-125(Ti) ratios of 2:2 and 3:2 are optimal to simultaneously exfoliate g-CN and to generate porous TiO_2_. Finally, the pore volumes and pore sizes were determined according to the Barrett–Joyner–Halenda (BJH) method (Figure 6b, Table 2). As previously, the higher pore volume values were obtained for the g-CN/TiO_2_ 2:1 and 3:1 materials (up to 0.29 cm^3^g^−1^). The large size of these pores further confirms the presence of mesopores. The high specific surface areas of g-CN/TiO_2_ 2:1 and 3:1 materials associated to their mesoporosity will provide more surface-active sites and thus should improve their photocatalytic performance.

**Figure 7:**
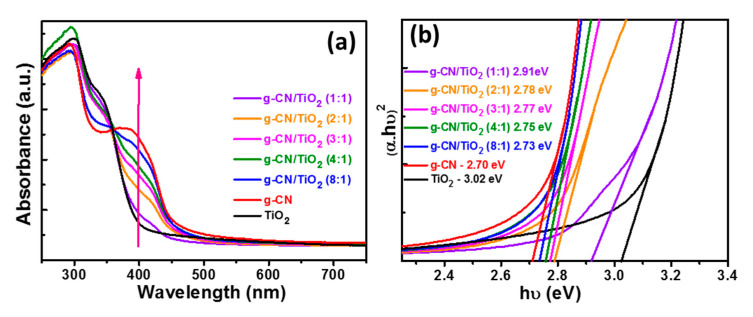
(**a**) UV-visible absorption spectra of TiO_2_, g-CN, and g-CN/TiO_2_ composites and (**b**) plots of (αhγ)^2^ vs hγ used to determine the energy bandgap of the photocatalysts.

UV-visible absorption spectra of TiO_2_, g-CN, and g-CN/TiO_2_ composites are shown in Figure 7a and the bandgap energy of the materials were determined using the relation αhʋ = A(hʋ − Eg)^2^ where α, h, ʋ, A, and Eg at the absorption coefficient, the Plank’s constant, the frequency of light, a constant, and the bandgap energy, respectively. Pure anatase TiO_2_ and g-CN exhibit bandgap energies of 3.02 and 2.70 eV, respectively, values in good accordance with those described in the literature (Figure 7b) [15,16,57]. With the increase of the g-CN loading in the photocatalysts, the UV-visible absorption in the 400–470 nm range increases and simultaneously the bandgap energy decreases from 3.02 to 2.73 eV. This extension of TiO_2_ absorption should improve its photocatalytic performance in the visible range.

### 3.2. Photodegradation of Pollutants under Visible Light Irradiation

First, the surface charges of g-CN and of the g-CN/TiO_2_ (3:1) catalysts in water were determined. For pure g-CN, the point of zero charge (pzc) was determined to be 4.23, value in good agreement with previous reports (Figure S7) [58]. As the pzc of pure TiO_2_ is usually between 3 and 4 [59], the pzc of the g-CN/TiO_2_ composite slightly decreases to 3.70 after deposition of TiO_2_ at the surface of g-CN sheets. These results show that the g-CN/TiO_2_ photocatalyst exhibits a positive charge at neutral pH and should associate via electrostatic interactions with negatively-charged pollutants in aqueous solution.

**Figure 8:**
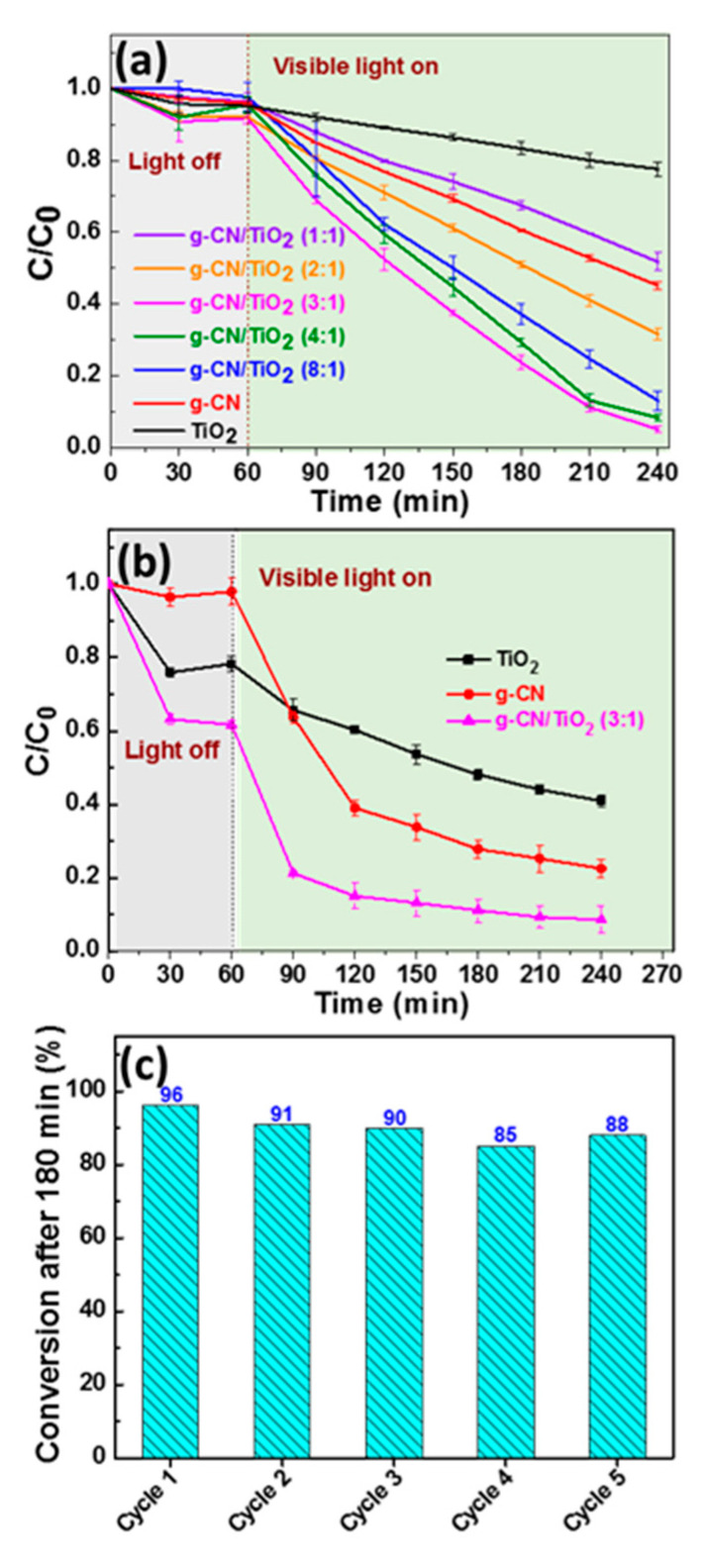
Photocatalytic degradation profiles of (**a**) Orange II and (**b**) tetracycline over TiO_2_, g-CN, and g-CN/TiO_2_ catalysts under visible light irradiation, and (**c**) photodegradation stability of Orange II using the g-CN/TiO_2_ (3:1) catalyst.

In a first set of experiments, the photocatalytic activity of g-CN/TiO_2_ materials was evaluated in the bleaching of the anionic diazo Orange II dye (concentration of 10 mgL^−1^) under visible light irradiation (intensity of 15 mWcm^−2^). Figure 8a depicts the variation of the relative concentration (C/C_0_) of Orange II vs irradiation time with respect to the changes of the dye absorption at 485 nm. The g-CN/TiO_2_ (3:1) composite exhibits the highest degradation efficiency for Orange II (ca. 95% bleaching after 180 min irradiation) while control experiments using TiO_2_ or g-CN showed significantly lower degradation efficiencies (22 and 50%, respectively) after the same illumination period. The photocatalytic activity increases with the increase of the g-CN/TiO_2_ ratio until 3:1 and then slightly decreases for composites prepared with g-CN:TiO_2_ ratios of 4:1 and 8:1, which suggest that the charge-carrier transfer efficiency is the highest when using the 3:1 g-CN:TiO_2_ ratio. The decrease of the photocatalytic efficiency for g-CN/TiO_2_ 4:1 and 8:1 composites is likely related to the decrease of their specific surface area as shown in Table 2 and to the increased charge carrier recombination (see below). Figure S8 shows the time dependent changes in the UV-visible absorption spectrum of Orange II during its photodegradation by the g-CN/TiO_2_ (3:1) catalyst. In the meantime, the orange color of the solution gradually disappeared, indicating that the structure of the dye was decomposed. The total organic carbon (TOC) decreased from 5.48 to 1.13 gL^−1^ after 180 min irradiation, which confirms the efficient mineralization of the dye. The first-order kinetics of Orange II degradation over the different photocatalysts are plotted in Figure S9. The apparent first-order rate constants k are 0.0033, 0.0058, 0.0156, 0.0137, and 0.0106 min^−1^ for g-CN/TiO_2_ catalysts prepared with g-CN:TiO_2_ ratios of 1:1, 2:1, 3:1, 4:1, and 8:1, respectively, further confirming that the g-CN/TiO_2_ (3:1) composite exhibits the highest photocatalytic activity.

The g-CN/TiO_2_ (3:1) photocatalyst was also successfully used for the degradation of tetracycline hydrochloride, an antibiotic commonly used in human and veterinary medicine [60] and which poses serious threats on various eco-systems due to its poor biodegradability [61]. The photocatalytic activity of the g-CN/TiO_2_(3:1) composite was compared to that of pure TiO_2_ and g-CN (Figure 8b). A marked absorption of tetracycline at the surface of g-CN/TiO_2_ and TiO_2_ catalysts (ca. 40 and 20%, respectively) was observed. Although tetracycline exists in its zwitterionic form at neutral pH [62], these results suggest that the enolate form of tetracycline strongly binds to the positively-charged surface of TiO_2_ and g-CN/TiO_2_ catalysts. The photocatalytic activity of the g-CN/TiO_2_ composite under visible light irradiation is significantly higher than that of TiO_2_ and g-CN, further confirming that the association of g-CN and TiO_2_ improves the separation of electron-hole pairs. The apparent first-order rate constant k determined for TiO_2_, g-CN, and g-CN/TiO_2_ photocatalysts are 0.0035, 0.0078, and 0.0093 min^−1^, respectively, and confirm that the kinetic of degradation is the highest for the g-CN/TiO_2_ (3:1) composite (Figure S10). As can also be seen from Figure S11, almost 95% of tetracycline is decomposed after 180 min visible light irradiation. Moreover, the TOC value decreased from 5.48 to 1.63 g L^−1^, further demonstrating that the g-CN/TiO_2_ (3:1) catalyst is of high interest for environmental applications. The performances of the g-CN/TiO_2_ (3:1) composite are higher than those of g-CN based catalysts recently developed for the degradation of TC (P- and S-doped g-CN, C-doped g-CN, or WO_3_/g-CN/Bi_2_O_3_, 70–85% degradation after ca. 1 h using a light intensity of 100 mWcm^−2^) when considering that a much lower intensity irradiation was used in our experiments (25 mWcm^−2^) [63,64,65].

The photocatalytic stability of the g-CN/TiO_2_ (3:1) composite was evaluated by five consecutive tests using Orange II as model pollutant (Figure 8c). A slight decrease of the dye degradation efficiency is observed after the first cycle (91%) but after the catalyst activity remains almost stable (88% after the fifth reuse), which indicates that the g-CN/TiO_2_ (3:1) composite shows a good stability as a photocatalyst. In addition, XRD analyses indicate that the crystallinity of the material is not affected by the reuses (Figure S12).

### 3.3. Active Species Involved in the Photodegradation, Photoelectrochemical Measurements, and Mechanism

**Figure 9:**
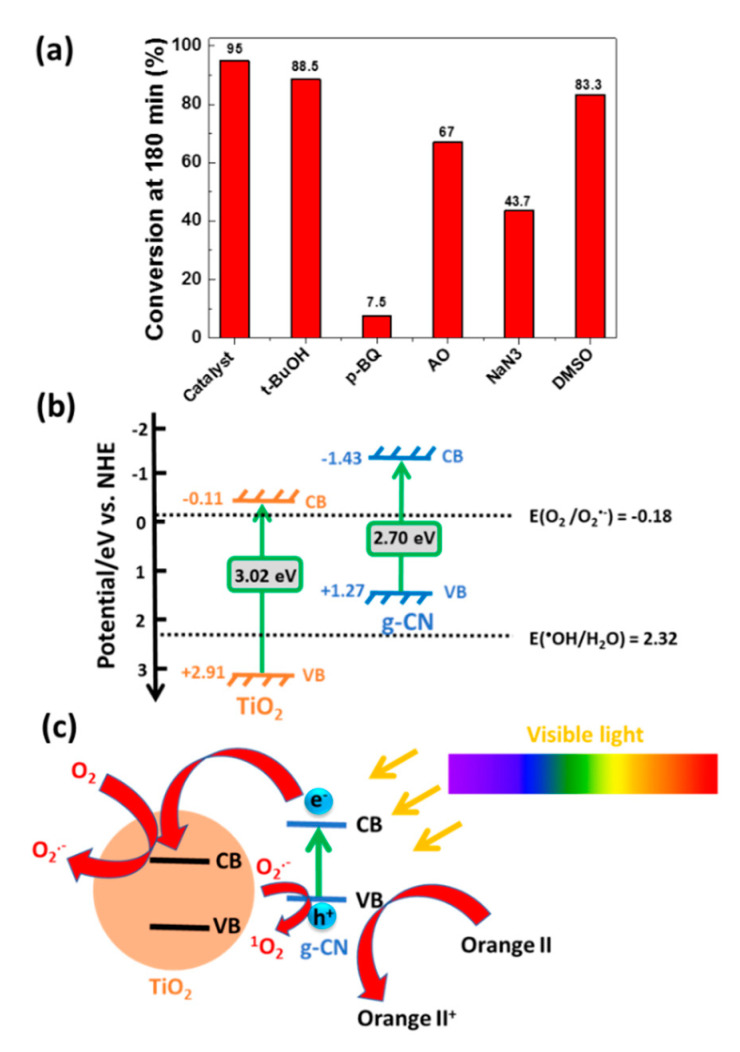
(**a**) Photodegradation of Orange II under visible light irradiation using the g-CN/TiO_2_ catalyst in the presence of reactive species trapping compounds, (**b**) band structure of the g-CN/TiO_2_ photocatalyst, and (**c**) schematic illustration of the delocalization of charge carriers and of the formation of reactive species involved in the photodegradation of Orange II.

To identify the active species responsible for the degradation of Orange II, trapping experiments were conducted using *tert*-butanol (*t*-BuOH), *p*-benzoquinone (*p*-BQ), ammonium oxalate (AO), sodium azide (NaN_3_), and DMSO as hydroxyl radicals (^●^OH), superoxide radicals (O_2_^●−^), holes (h^+^), singlet oxygen (^1^O_2_), and electron (e^-^) scavengers, respectively [15,16]. Figure 9a shows the influence of these scavengers on the photocatalytic performance of the g-CN/TiO_2_ (3:1) composite after 180 min irradiation. Weak decreases of the Orange II photodegradations were observed when adding *t*-BuOH and DMSO (88.5 and 83.3%, respectively) indicating that ^●^OH radicals and electrons only play a minor role in the mechanism. The photodegradation efficiencies more significantly declined upon addition of AO and NaN_3_ (67 and 43.7%, respectively) indicating that h^+^ and ^1^O_2_ are involved in the degradation. Finally, O_2_^●−^ radicals play the key role in the photodegradation as the most deleterious effect was observed using *p*-BQ (7.5% after 180 min irradiation).

The band edge position of the valence band (VB) of TiO_2_ at the point of zero charge can be calculated using the empirical Equation (2):

*E_CB_* = *χ* − *E^e^* − *0.5 Eg* (2)

where *E_CB_* is the CB potential, *χ* is the absolute electronegativity of TiO_2_ (5.90 eV), *E^e^* is the energy of free electrons on the hydrogen scale (ca. 4.5 eV), and *Eg* is the bandgap energy (3.02 eV) [66]. The band edge positions of the VB and of the CB of TiO_2_ nanoparticles were estimated to be +2.91 and −0.11 eV, respectively. For g-CN with a *χ* value of 4.42 eV, E_CB_ and E_VB_ values were determined to be −1.43 and +1.27 eV, respectively. A diagram showing the band structure of g-CN/TiO_2_ nanocomposite is presented in Figure 9b along with the redox potentials of the reference reactions.

On the basis of these data, a scheme for the photogenerated electron/hole transfer steps under visible light irradiation at the interface of the g-CN/TiO_2_ catalyst can be proposed (Figure 9b,c). Under visible light irradiation (λ > 420 nm), only g-CN is able to absorb photons, which results in the transfer of electrons from the VB to the CB of TiO_2_. Due to the electrostatic field at the junction, photoexcited electrons can easily transfer to the CB while holes remain in g-CN, thereby hindering the charge carriers recombination and increasing the lifetimes of photogenerated electrons and holes. Then, separated electrons and holes can initiate reduction and oxidation reactions with O_2_ and H_2_O molecules adsorbed on the catalyst surface. The VB potential of g-CN (+1.27 eV) is higher than that of the ^●^OH/H_2_O couple (+2.32 eV) and holes in the VB g-CN cannot react with H_2_O to generate ^●^OH radicals. However, these holes can oxidize Orange II into Orange II^+^. In the meantime, electrons accumulated in the CB of TiO_2_ can easily react with O_2_ to generate O_2_^●−^ radicals. These radicals can directly oxidize the dye or transfer electrons to the holes in the VB of g-CN to give ^1^O_2_. These data are in good accordance with scavenging results described in Figure 9a where O_2_^●−^, holes and ^1^O_2_ were demonstrated to play the major role in the photodegradation mechanism.

**Figure 10:**
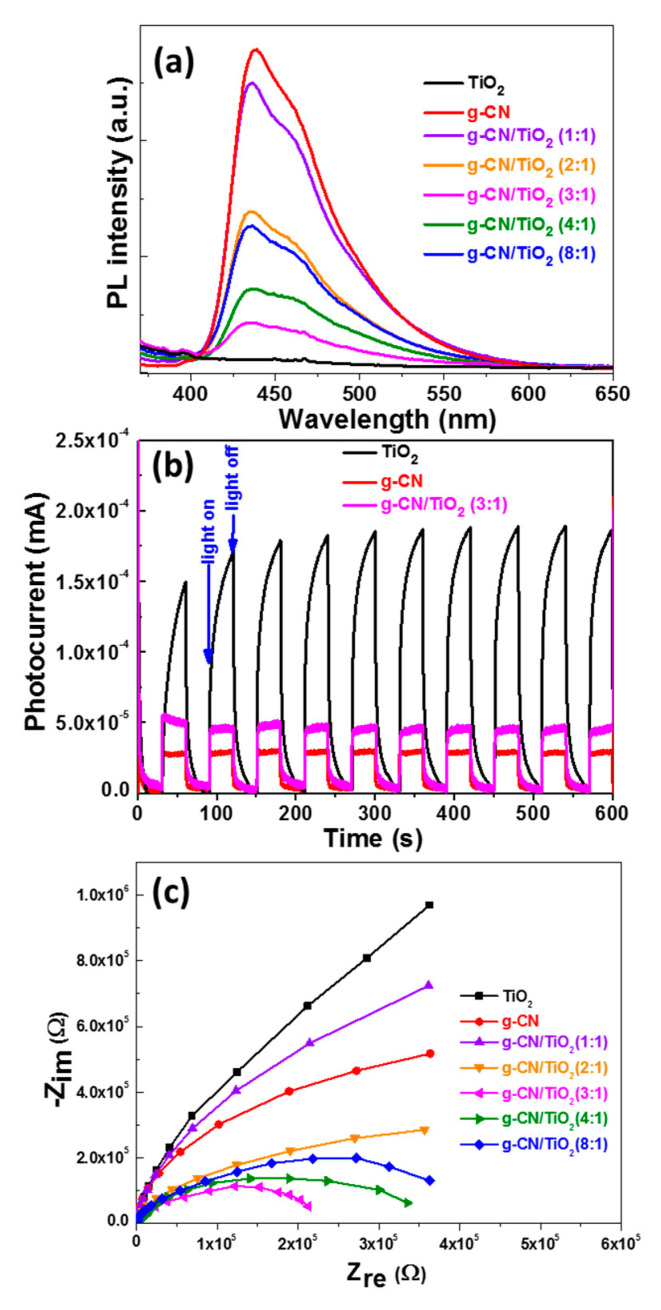
(**a**) Room temperature photoluminescence (PL) emission spectra of TiO_2_, g-CN, and g-CN/TiO_2_ materials (λ_ex_ = 350 nm), (**b**) Photocurrent responses of TiO_2_, g-CN, and g-CN/TiO_2_ (3:1) composite under visible light irradiation and (**c**) EIS Nyquist plots of TiO_2_, g-CN, and g-CN/TiO_2_ materials.

To support the enhanced photocatalytic performance of the g-CN/TiO_2_ (3:1) composite under visible light irradiation, the recombination process of photogenerated electron-hole pairs in g-CN, TiO_2_ and g-CN/TiO_2_ composites was first investigated by PL spectroscopy. Upon excitation at 350 nm, g-CN exhibits a strong PL emission with the main signal located at 439 nm while TiO_2_ is non-fluorescent (Figure 10a). The shape of the PL peak for g-CN/TiO_2_ composites is similar to that of g-CN but its intensity is weaker indicating that the electron-hole pair’s recombination is hampered. There is no correlation between the g-CN loading in g-CN/TiO_2_ composites and the PL intensity gradually decreases when increasing the g-CN:TiO_2_ ratio from 1:1 to 3:1 before re-increasing for the 4:1 and 8:1 ratios. The separation of electron-hole pairs is the most efficient in the g-CN/TiO_2_ (3:1) material, which is consistent with photocatalytic results.

The electronic interactions in the various g-CN/TiO_2_ photocatalysts were also studied by measuring the photocurrent responses and by using electrochemical impedance spectroscopy (EIS). Figure 10b shows the I–t curves of g-CN, TiO_2_, and g-CN/TiO_2_ (3:1) materials during ten on–off cycles of visible light irradiation. The photocurrent quickly increases when light is turned on and decreases to zero when the light is turned off, indicating that the materials respond to light with a good reproducibility. An improved charge carrier separation efficiency and a fastest charge transfer through the electrode interface are observed for the g-CN/TiO_2_ (3:1) composite as the photocurrent response is ca. 1.8-fold higher than that of g-CN. Due to its bandgap energy of 3.02 eV (410 nm), pure TiO_2_ responds to visible light irradiation. However, the photocurrent continuously increases during the illumination and does not reach a steady state contrary to g-CN and to the g-CN/TiO_2_ (3:1) composite. A similar behavior was previously observed and may originate either from photo-excited states exhibiting a short lifetime and/or from a better contact between TiO_2_ and the electrode during photoelectrochemical measurements [19].

The transfer resistance of electrons on the electrode surface plays also a key role on the electron transfer efficiency. The Nyquist plots of the EIS for TiO_2_, g-CN, and g-CN/TiO_2_ photocatalysts are shown in Figure 10c. The smallest diameter of the semi-circular Nyquist plots is observed for the g-CN/TiO_2_ (3:1) composite which further confirms that the electron transfer is the fastest in this material.

### 3.4. Hydrogen Photoproduction

The hydrogen production activity of g-CN/TiO_2_/NiS catalysts was evaluated using a 300 W Xenon lamp as light source and an aqueous solution containing 10 vol.% glycerol as sacrificial substrate. Pure TiO_2_ and g-CN were used as references in these experiments. The efficient and cheap NiS co-catalyst which has shown its effectiveness for H_2_ photoproduction using g-CN and TiO_2_ was used instead of noble metals like Pt or Pd [67,68]. After deposition of NiS at the surface of g-CN/TiO_2_ (4:1), (3:1), and (2:1) composites, photogenerated electrons likely transfer from the g-CN/TiO_2_ photocatalyst to the valence band of the NiS semiconductor (Z-scheme transfer) which promotes H_2_ evolution according to Equations (3) and (4):

NiS + e^−^ + H^+^ → HNiS (3)

HNiS + e^−^ + H^+^ → NiS + H_2_ (4)

Meanwhile, electrons are reinjected in the g-CN/TiO_2_ photocatalyst via the oxidation of the sacrificial substrate.

**Figure 11:**
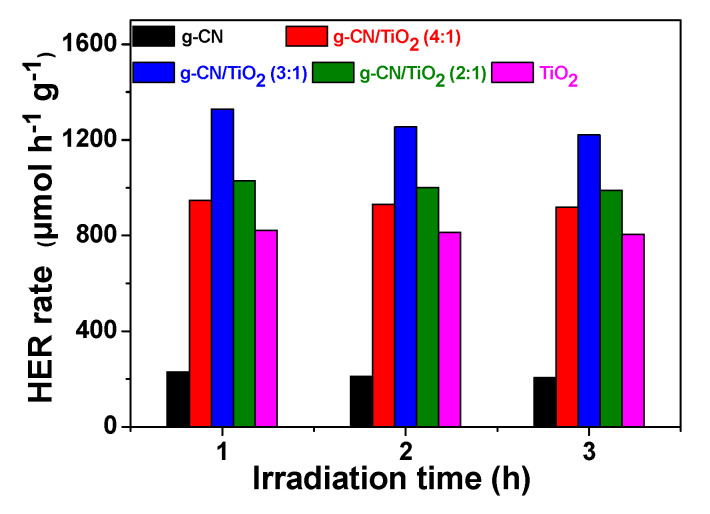
Photocatalytic hydrogen production rates after 1, 2, and 3 h illumination of g-CN, TiO_2_, and g-CN/TiO_2_ catalysts associated to the NiS cocatalyst (1.5 wt.%) using a 300 W Xe lamp as irradiation source and water containing 10 vol.% glycerol as sacrificial substrate.

Control experiments show that H_2_ is not produced in the absence of the photocatalyst or light irradiation. As can be seen from Figure 11, H_2_ is produced immediately after turning on light and the hydrogen evolution rate (HER) remains almost stable along the 3 h of irradiation. The highest HER values were obtained for the g-CN/TiO_2_ (3:1) catalyst (1330, 1256, and 1223 μmolh^−1^g^−1^ after 1, 2, and 3 h of irradiation, respectively). These HERs are ca. 1.65- and 5.8-times higher than those measured for TiO_2_ and g-CN. The apparent quantum efficiency (AQE) of the g-CN/TiO_2_ (3:1) photocatalyst is of 0.22%, value 1.7-fold and 5.8-fold higher than those determined for pure TiO_2_ and g-CN, respectively. These results further demonstrate that the heterojunction constructed between TiO_2_ and g-CN combined to the high specific surface of the catalyst contribute to the enhancement of photogenerated electron transfer and thus to the photocatalytic activity. Finally, the photocatalytic activity for H_2_ production is at least 2.6-times higher than that of binary g-CN/TiO_2_ catalyst recently described in the literature (the highest HER values vary from 446 to 500 μmolh^−1^g^−1^) [18,25,32,33]. Noteworthy is also that some higher values were reported (up to 8931 μmolh^−1^g^−1^) but using Pt as co-catalyst [22].

## 4. Conclusions

Photocatalysts associating TiO_2_ nanoparticles with an average size of ca. 12 nm and g-CN sheets were efficiently prepared by thermolysis of MIL-125(Ti) particles in the presence of g-CN. The high specific surface area of MIL-125(Ti) is transferred to TiO_2_ nanoparticles and g-CN/TiO_2_ composites exhibiting specific surface area up to 102 m^2^g^−1^ could be engineered using the synthetic protocol developed in this work. The g-CN/TiO_2_ (3:1) composite was demonstrated to exhibit the highest photocatalytic activity both for the degradation of pollutants like Orange II or tetracycline and for hydrogen production (HER of 1330 μmolh^−1^g^−1^ after 1 h irradiation without using Pt as co-catalyst). The close interfacial connection between TiO_2_ and g-CN allows the efficient charge carriers separation with photo-excited electrons transferred from g-CN to TiO_2_ while holes remain in g-CN. The design of heterostructured materials using the thermal decomposition of metal organic frameworks to generate porous metal oxide like TiO_2_ was demonstrated to be an efficient strategy to improve the solar utilization for photocatalytic applications.

## Figures and Tables

**Table 1:** Theoretical and actual g-CN:TiO_2_ ratios determined by thermogravimetric analysis (TGA) analyses.

Theoretical g-CN:TiO_2_ Ratio	1:1	2:1	3:1	4:1	8:1
Actual g-CN:TiO_2_ Ratio	0.28:1	0.91:1	1.59:1	2.12:1	4.41:1

**Table 2:** Brunauer–Emmett–Teller (BET) specific surface areas, pore volumes and pore sizes of g-CN, TiO_2_, and g-CN/TiO_2_ composites.

Sample	BET (m^2^g^−1^)	Pore Volume (cm^3^g^−1^)	Pore Size (nm)
g-CN	9.8 ± 0.2	0.07	17.75
TiO_2_	56.3 ± 0.4	0.18	10.19
g-CN/TiO_2_ (1:1)	59.8 ± 0.3	0.20	11.15
g-CN/TiO_2_ (2:1)	102.2 ± 0.4	0.29	9.42
g-CN/TiO_2_ (3:1)	86.2 ± 0.3	0.23	9.53
g-CN/TiO_2_ (4:1)	63.8 ± 0.1	0.20	11.19
g-CN/TiO_2_ (8:1)	48.8 ± 0.2	0.21	16.05

## Supplementary Materials

The following are available online at https://www.mdpi.com/2079-4991/10/7/1387/s1, Figure S1: (a) SEM image, (b) EDX analysis, and (c–f) EDX mapping of the g-CN/MIL-125(Ti) (3:2) composite. Figure S2: (a) SEM image, (b) EDX analysis, and (c-g) EDX mapping of the g-CN/TiO_2_ (3:1) composite. Figure S3. XRD analysis of the (002) peak for (a) bulk g-CN and (b) exfoliated g-CN using the DIFFRAC.EVA software from Bruker. Figure S4. XRD patterns of bulk and exfoliated g-CN. Figure S5: XPS survey spectrum of the g-CN/TiO_2_ (3:1) photocatalyst. Figure S6: High resolution XPS spectra of (a) C 1s, (b) N 1s, (c) Ti 2p, and (d) O 1s for the g-CN/TiO2 (3:1) photocatalyst. Figure S7: Zeta potentials of g-CN and g-CN/TiO_2_ (3:1) photocatalysts as a function of pH. Figure S8: UV-vis spectrum changes of Orange II during its photodegradation by the g-CN/TiO_2_ (3:1) composite. Figure S9: Pseudo-first-order kinetics fitted curves of Orange II degradation over TiO_2_, g-CN, and g-CN/TiO_2_ composites under visible light irradiation. Figure S10: Pseudo-first-order kinetics fitted curves of tetracycline degradation over TiO_2_, g-CN, and the g-CN/TiO_2_ (3:1) composite under visible light irradiation. Figure S11: UV-vis spectrum changes of tetracycline during its photodegradation by the g-CN/TiO_2_ (3:1) composite. Figure S12: XRD patterns of the g-CN/TiO_2_ (3:1) catalyst after synthesis (black line) and after 5 reuses for the degradation of the Orange II dye (red line).
