# Supplementary material for: Heterostructured g-CN/TiO2 Photocatalysts Prepared by Thermolysis of g-CN/MIL-125(Ti) Composites for Efficient Pollutant Degradation and Hydrogen Production
Source: Nanomaterials (Basel). 2020 Jul 16;10(7):1387. doi: 10.3390/nano10071387 (PMC7407120; doi:10.3390/nano10071387)
Supplement: Supplementary file 1 [file nanomaterials-10-01387-s001.pdf]

ELECTRONIC SUPPORTING INFORMATION

Heterostructured g-CN/TiO_2 Photocatalysts Prepared by Thermolysis of g-CN/MIL-125(Ti) Composites for Efficient Pollutant Degradation and Hydrogen Production

Batukhan Tatykayev [1,2], Bilel Chouchene [1], Lavinia Balan [3], Thomas Gries [4], Ghouti Medjahdi [4], Emilien Girot [1], Bolat Uralbekov [2] and Raphaël Schneider [1,*]

[1] Université de Lorraine, CNRS, LRGP, F-54000 Nancy, France; b.tatykayev@gmail.com (B.T.); bilel.chouchene@univ-lorraine.fr (B.C.); emilien.girot@univ-lorraine.fr (E.G.)

[2] Department of General and Inorganic Chemistry, Al-Farabi Kazakh National University, Al-Farabi Av., 71, Almaty 050040, Kazakhstan; bulat.ural@gmail.com

[3] Conditions Extrêmes et Matériaux : Haute Température et Irradiation, UPR 3079 CNRS, Site Haute Température, 1D avenue de la Recherche Scientifique, 45071 Orléans, France; lavinia.balan@cnrs-orleans.fr

[4] Université de Lorraine, CNRS, IJL, F-54000 Nancy, France; thomas.gries@univ-lorraine.fr (T.G.); ghouti.medjahdi@univ-lorraine.fr (G.M.)

* Correspondence: raphael.schneider@univ-lorraine.fr; Tel.: +33-3 78 74 37

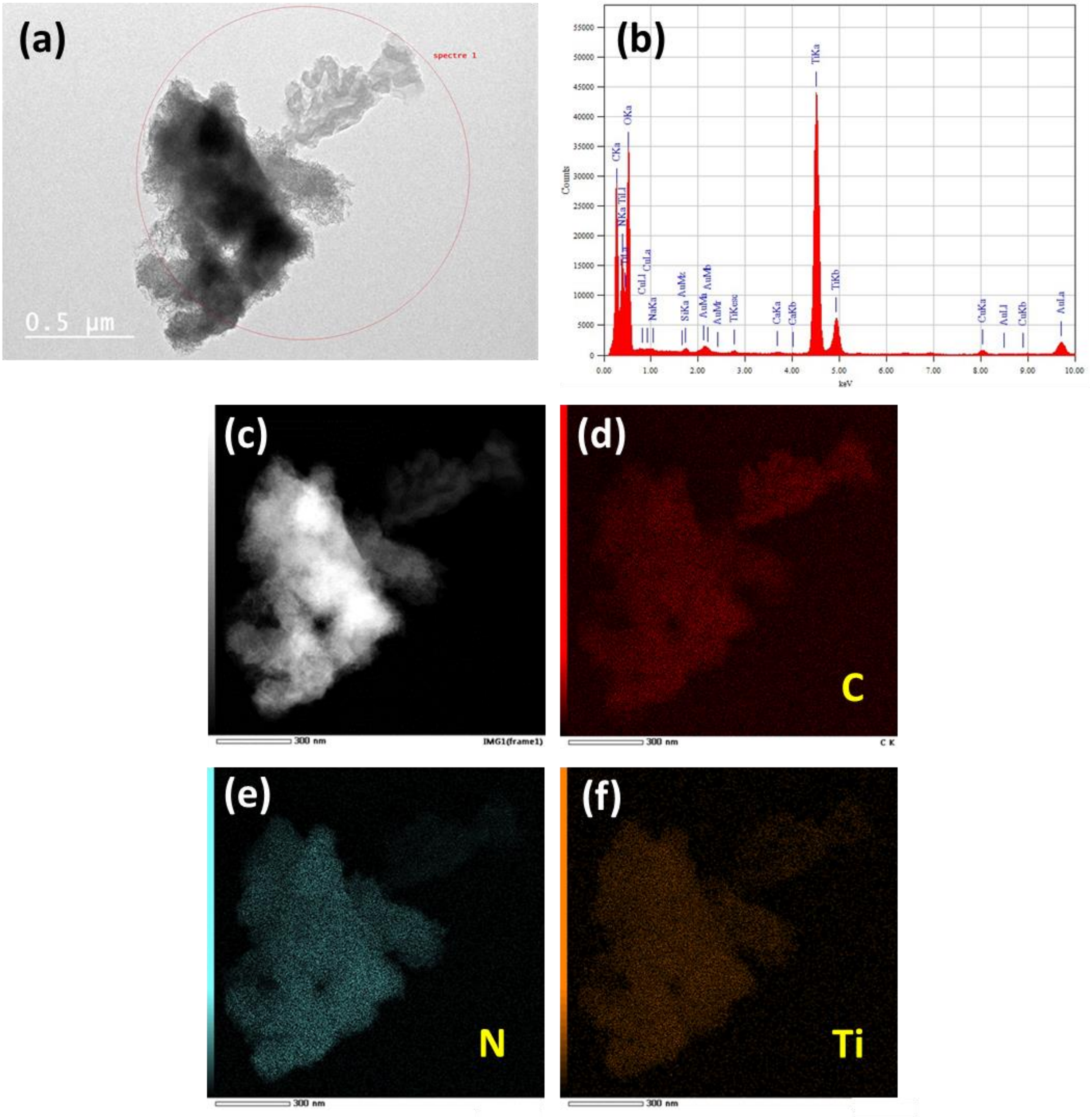

Figure S1. (a) SEM image, (b) EDX analysis and (c-f) EDX mapping of the g-CN/MIL-125(Ti) (3 :2) composite.

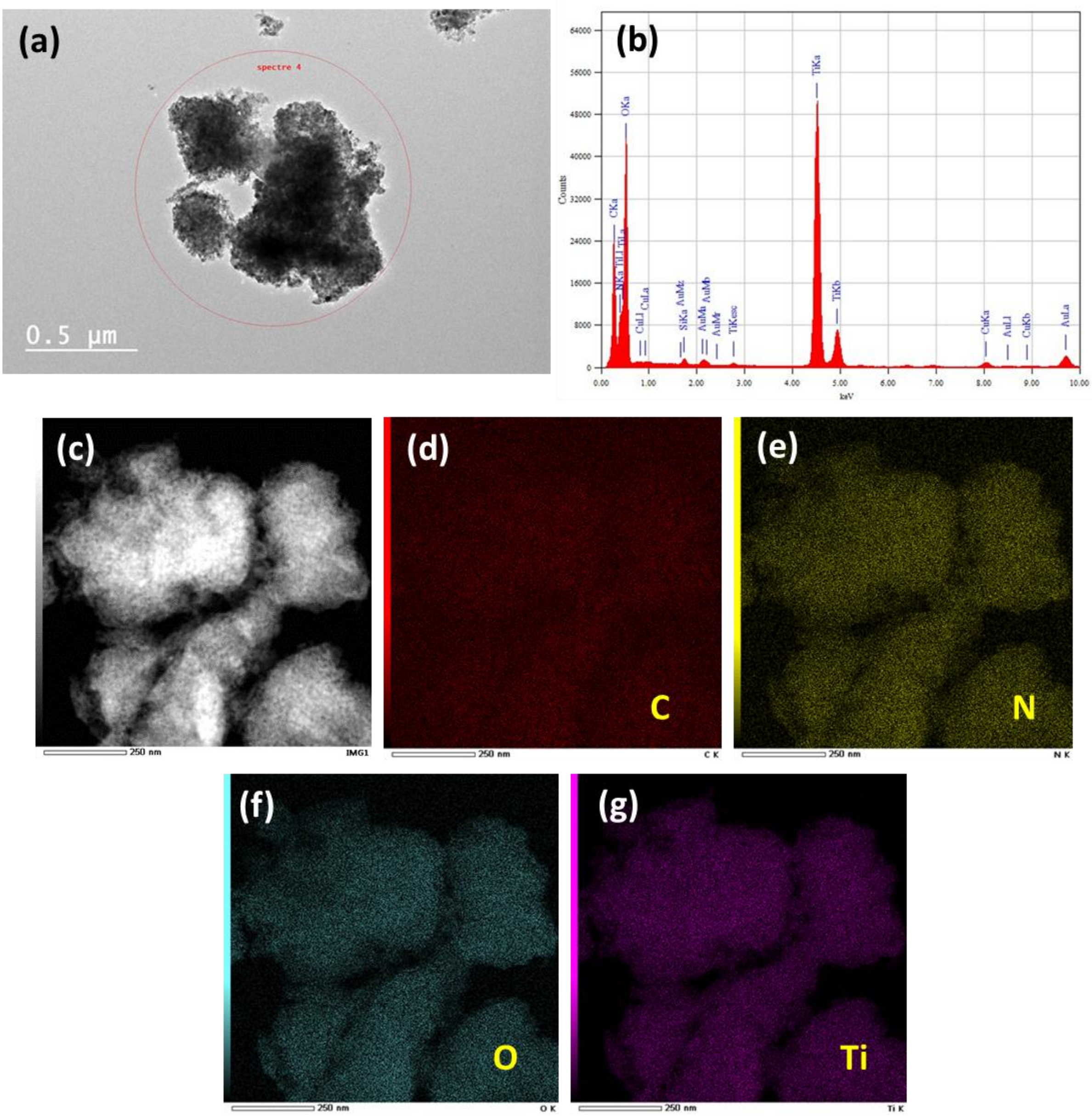

Figure S2. (a) SEM image, (b) EDX analysis and (c-g) EDX mapping of the g-CN/TiO_2 (3:1) composite.

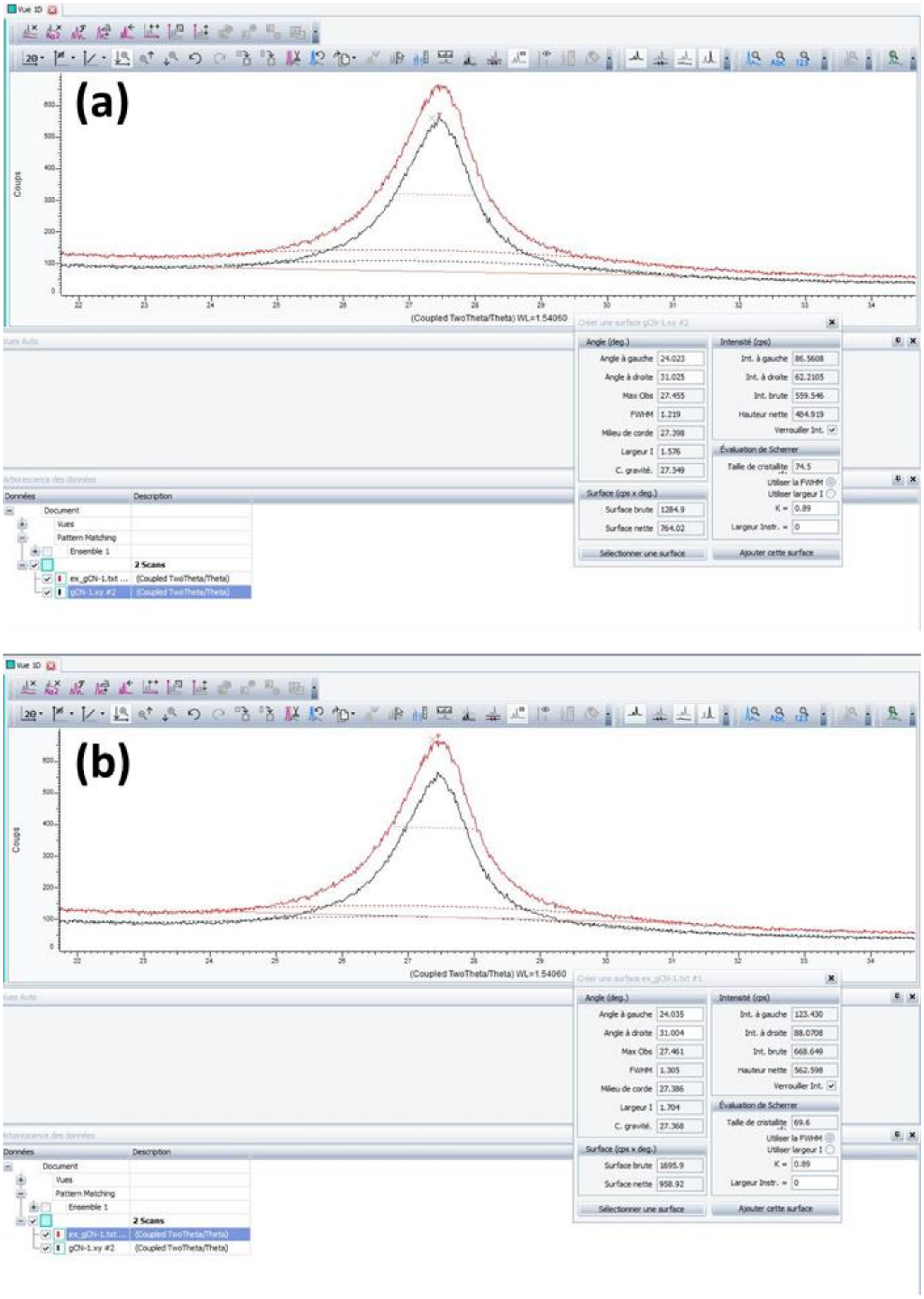

Figure S3. XRD analysis of the (002) peak for (a) bulk g-CN and (b) exfoliated g-CN using the DIFFRAC.EVA software from Bruker.

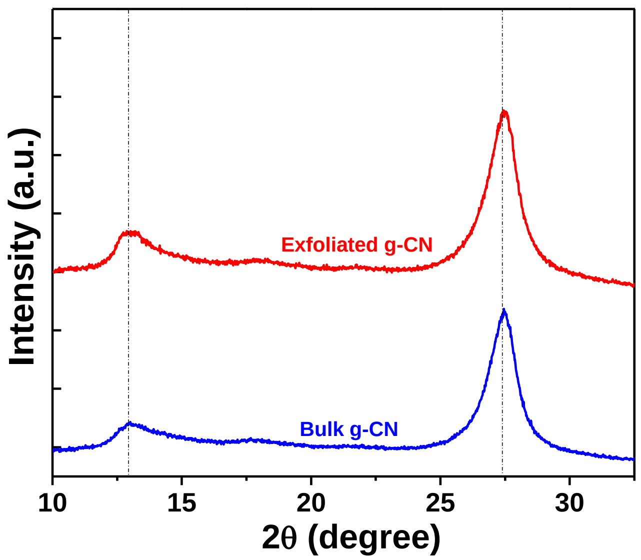

Figure S4. XRD patterns of bulk and exfoliated g-CN.

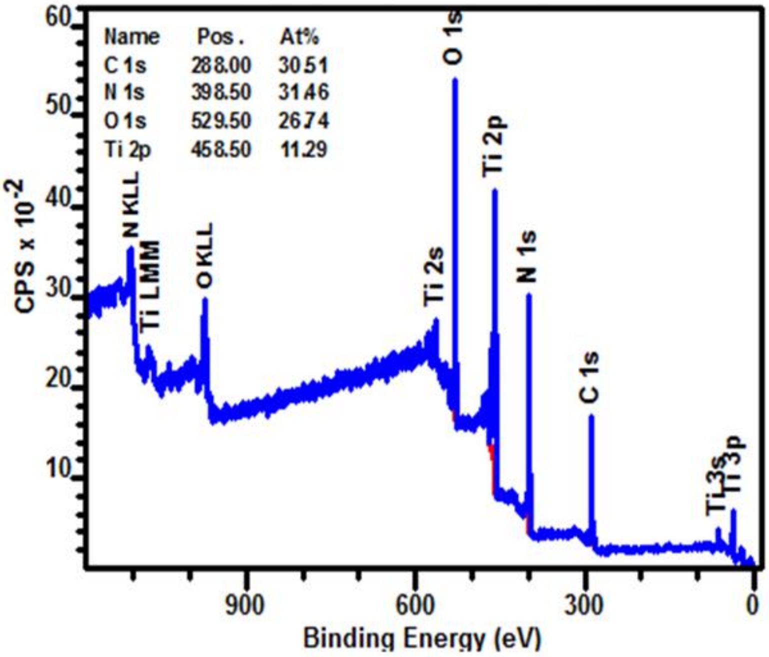

Figure S5. XPS survey spectrum of the g-CN/TiO_2 (3:1) photocatalyst.

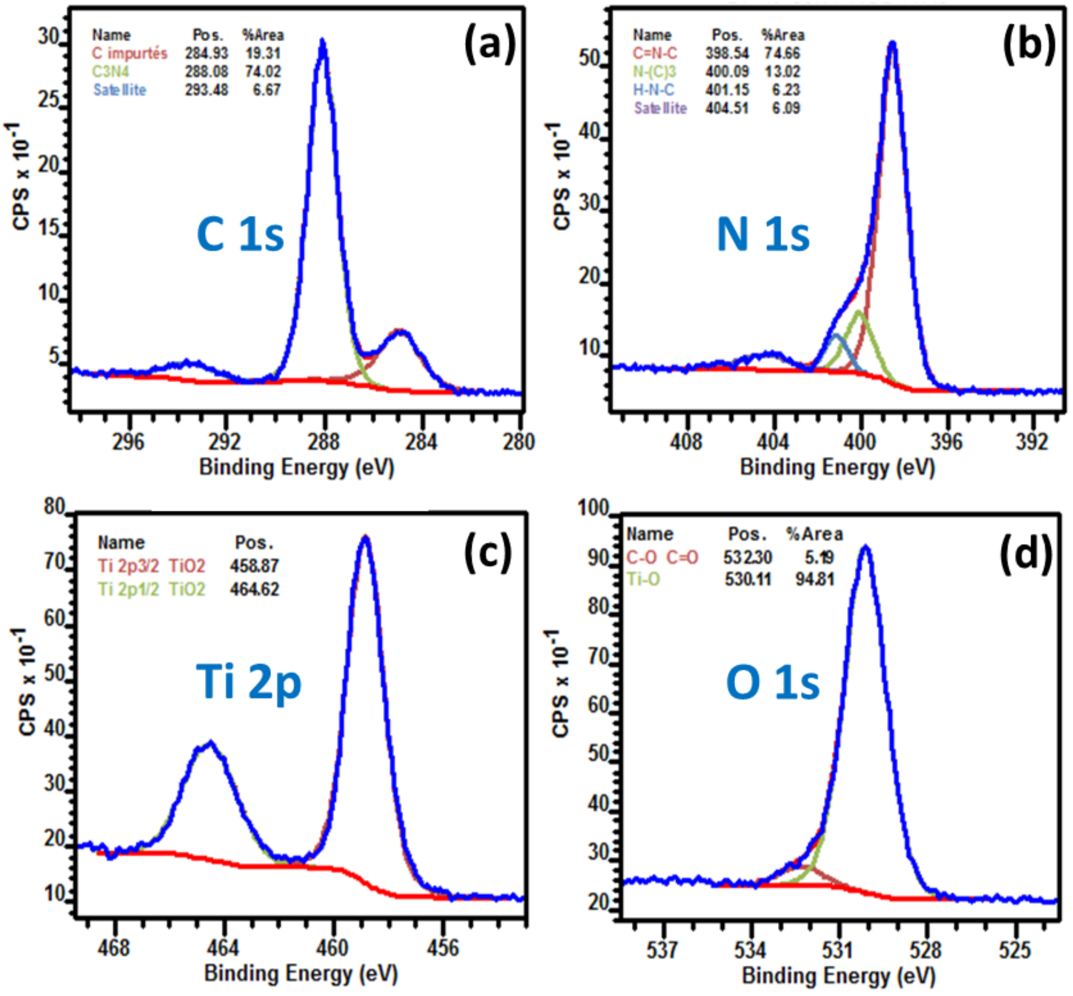

Figure S6. High resolution XPS spectra of (a) C 1s, (b) N 1s, (c) Ti 2p and (d) O 1s for the g-CN/TiO2 (3:1) photocatalyst.

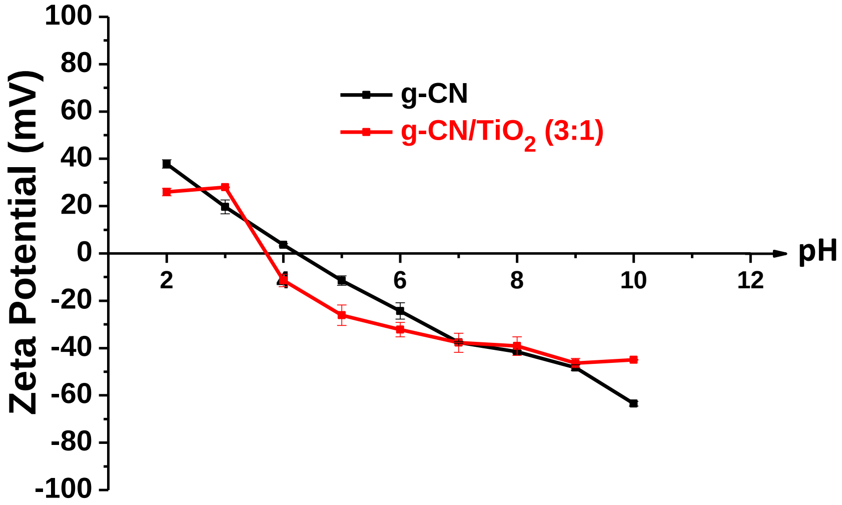

Figure S7. Zeta potentials of g-CN and g-CN/TiO_2 (3:1) photocatalysts as a function of pH.

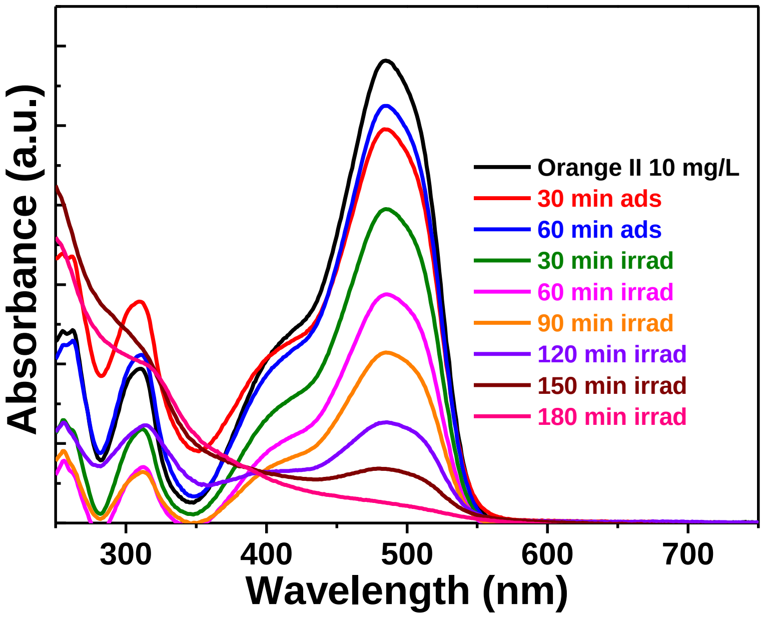

Figure S8. UV-vis spectrum changes of Orange II during its photodegradation by the g-CN/TiO_2 (3:1) composite.

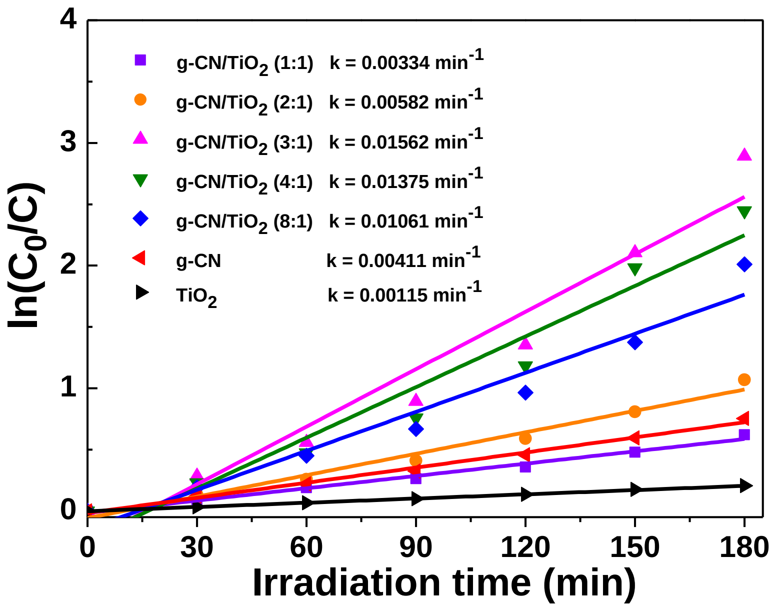

Figure S9. Pseudo-first-order kinetics fitted curves of Orange II degradation over TiO_2, g-CN and g-CN/TiO_2 composites under visible light irradiation.

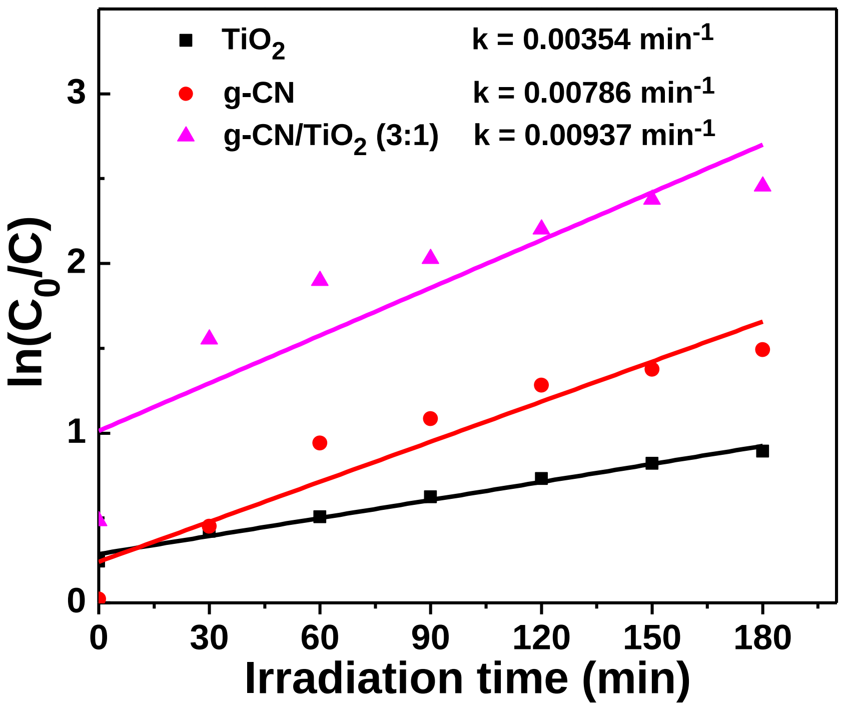

Figure S10. Pseudo-first-order kinetics fitted curves of tetracycline degradation over TiO_2, g-CN and the g-CN/TiO_2 (3:1) composite under visible light irradiation.

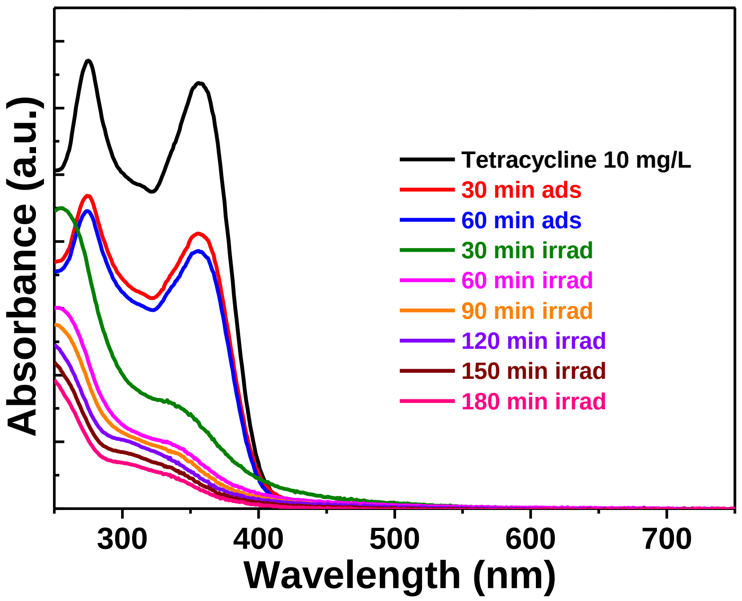

Figure S11. UV-vis spectrum changes of tetracycline during its photodegradation by the g-CN/TiO_2 (3:1) composite.

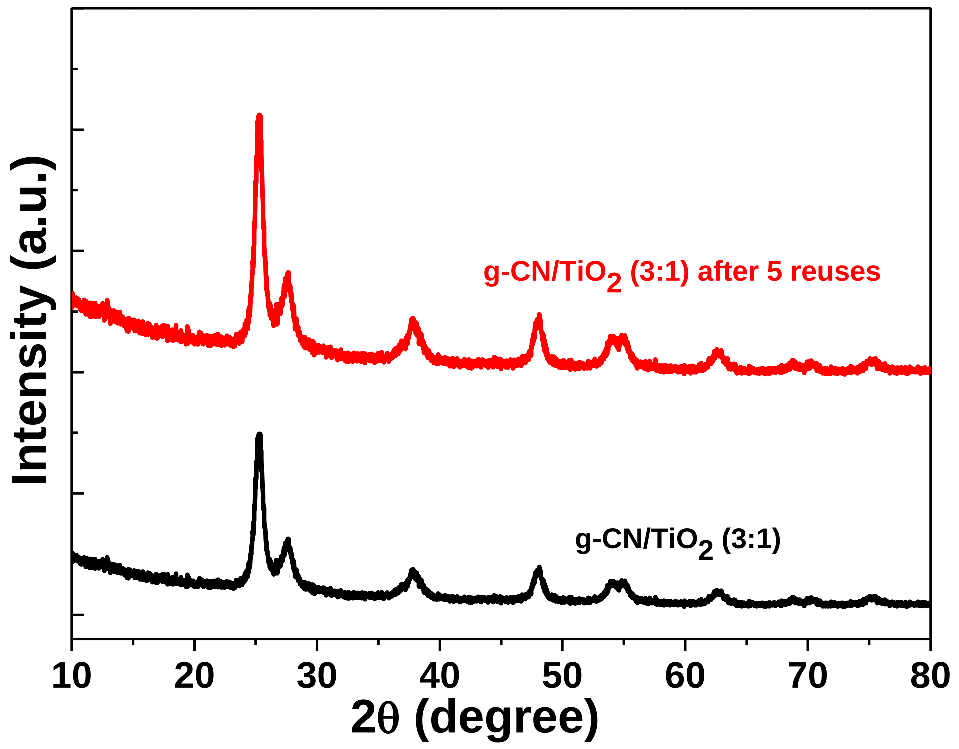

Figure S12. XRD patterns of the g-CN/TiO_2 (3:1) catalyst after synthesis (black line) and after 5 reuses for the degradation of the Orange II dye (red line).
